# Software-Defined Networking Solutions, Architecture and Controllers for the Industrial Internet of Things: A Review

**DOI:** 10.3390/s21196585

**Published:** 2021-10-01

**Authors:** Claudio Urrea, David Benítez

**Affiliations:** Electrical Engineering Department, Faculty of Engineering, University of Santiago of Chile, Las Sophoras 165, Estación Central, Santiago 9170124, Chile; david.benitez@usach.cl

**Keywords:** industrial internet of things communications, software-defined networking, communications performance optimization, comprehensive SDN solution

## Abstract

The use of Software-Defined Networking (SDN) in the communications of the Industrial Internet of Things (IIoT) demands more comprehensive solutions than those developed to date. The lack of an SDN solution applicable in diverse IIoT scenarios is the problem addressed in this article. The main cause of this problem is the lack of integration of a set of aspects that should be considered in a comprehensive SDN solution. To contribute to the solution of this problem, a review of the literature is conducted in this article, identifying the main requirements for industrial networks nowadays as well as their solutions through SDN. This review indicates that aspects such as security, independence of the network technology used, and network centralized management can be tackled using SDN. All the advantages of this technology can be obtained through the implementation of the same solution, considering a set of aspects proposed by the authors for the implementation of SDNs in IIoT networks. Additionally, after analyzing the main features and advantages of several architectures proposed in the literature, an architecture with distributed network control is proposed for all SDN network scenarios in IIoT. This architecture can be adapted through the inclusion of other necessary elements in specific scenarios. The distributed network control feature is relevant here, as it prevents a single fault-point for an entire industrial network, in exchange for adding some complexity to the network. Finally, the first ideas for the selection of an SDN controller suitable for IIoT scenarios are included, as this is the core element in the proposed architecture. The initial proposal includes the identification of six controllers, which correspond to different types of control planes, and ten characteristics are defined for selecting the most suitable controller through the Analytic Hierarchy Process (AHP) method. The analysis and proposal of different fundamental aspects for the implementation of SDNs in IIoT in this article contribute to the development of a comprehensive solution that is not focused on the characteristics of a specific scenario and would, therefore, be applicable in limited situations.

## 1. Introduction

Currently, the manufacturing sector and the industry in general have witnessed considerable advances, thanks to the advent of new technologies and the progress made in already existing ones [1,2]. In this way, today’s world has a more intelligent and interconnected industry, which has led to the Industrial Internet of Things (IIoT) and the increasingly commented on fourth industrial revolution, denominated as Industry 4.0. In addition to the new elements and advantages brought up by this industry, there are also different challenges [3,4], some of them associated with the communication networks of this new smart industry.

To face the challenges and new requirements for industrial communication networks, particularly in the IIoT context, new technological paradigms can be utilized. Software-Defined Networking (SDN) [5,6] is a novel technology that achieves more flexibility, management, and adaptability by separating control and traditional network planes. SDNs have been used as a possible solution to the new industrial communication requirements [7,8,9,10,11]. Therefore, the general objective of improving the performance of Industry 4.0 communication systems can be achieved through the development of SDN-based systems. Particularly, critical applications, such as the control of industrial robots, can benefit from this paradigm in the modern industry [12].

This article presents several aspects that can be improved on in the communications of any industry, mainly those under the IIoT paradigm. The enhancement of industrial communications, as a general objective, comprises other more concrete objectives. Some current demands of industrial communications, such as achieving adaptive transmission in a communication system or efficiency in terms of fault-tolerance, are topics addressed in the literature [13]. A number of these demands can be satisfied using SDN, despite the challenges faced by this technology in some scenarios. A single controller is not suitable for large scale networks; however, controller placement problems occur when adding multiple controllers to these networks [14]. Other challenges for the application of SDNs derive from their use in multi-hop scenarios [15] and the adequate scalability of the control plane [16]; therefore, currently SDN is not a perfect solution for all situations.

The literature contains some works that review several aspects necessary to achieve a dynamic and flexible control of communication networks in IIoT. An example of this is the research conducted in [17], which, after dealing with a set of general aspects on the use of SDN in IIoT, focuses on the implementation of two architectures in low power wireless network scenarios.

Several trends and challenges for communication networks in IIoT have aspects in common with Cyber-Physical Systems (CPS). SDNs have also been employed in CPS [18] to ensure flexibility and heterogeneity without compromising the Quality of Service (QoS) required. In turn, SDNs are employed to guarantee the resilience required in industrial/manufacturing systems and CPS in [19], since some operations should not be interrupted at any time.

Solutions that employ SDN in IIoT scenarios are generally quite specific. Difficulties such as adaptive transmission, fault tolerance, or security have been tackled through SDNs with new protocols [20], architectures with models that calculate redundant paths [21], and anomaly detection methods [22], to cite some examples. All these solutions target specific problems. In this sense, the development of a set of SDN applications that focuses on several limitations is timely and feasible. This would be a comprehensive solution in which a variety of applications would attend the main demands of the modern industrial communication systems, such as network reliability, adaptive transmission, fault tolerance, and real-time traffic.

The development of a comprehensive solution, one with SDN as its core element and that is applicable to IIoT scenarios, would improve traditional networks. Several researchers have indicated that the changes necessary to adapt these networks to the new requirements can be cumbersome. Some of the main difficulties in adapting traditional networks to the needs of the smart industry are scalability, flexibility, and delivery of a high enough QoS [23,24].

To develop this type of solution, a base architecture in which the different industrial, SDN, and technology elements integrated into the final solution are defined is necessary. With a well-established architecture, SDN applications that focus on the demands above can be developed, while other architectures may emerge thanks to the decoupling of the control and data planes mentioned.

Regarding the development of an industrial network model that meets the current demand, Radanliev et al. [25] state that the literature is missing a design process for integrating SDNs—constantly evolving systems—and technologies in a clear and understandable step by step model.

Therefore, the analysis of these solutions to identify the most valuable aspects of each is useful. The objective of this article is to define a set of main aspects to be considered in an SDN solution for IIoT, seeking to contribute to the development of a comprehensive SDN solution for diverse scenarios in IIoT. The contribution is fundamentally based on the review, identification, and analysis of a set of aspects fundamental to the development of a solution of this type and a proposal for using an architecture with distributed network control regardless of the type of scenario. Additionally, the first ideas for the selection of an SDN controller suitable for IIoT scenarios are included. To conduct this work, several articles were reviewed which addressed the integration of SDN into the Internet of Things (IoT), mainly in IIoT scenarios from different perspectives. In this way, the main aspects that require input from the scientific community were identified. Such contributions should not only focus on the solution of concrete technical aspects, but also on the development of a set of SDN applications capable of offering a comprehensive solution for industrial communications.

The article is structured as follows: in Section 2, some characteristics of the current communication networks in IIoT are analyzed, highlighting their limitations. Section 3 focuses on the use of SDN in IIoT; specifically, the way in which this technology may be the solution for many of these limitations is explained in Section 3.1. Section 3.2 addresses the integration of SDN into other technologies. Section 4.1 presents several aspects to consider in the development of a comprehensive SDN solution for IIoT. In Section 4.2, aspects related to the simulation and implementation of SDN-based networks in IIoT are explained. In Section 4.3, an architecture is proposed for the use of SDN in IIoT, and the selection of a SDN controller is addressed. Finally, Section 5 highlights the main aspects identified in this work.

## 2. Fundamental Aspects of Communication Networks in the IIoT

In this section, a group of features from communication networks in IIoT is presented, which derive from the intrinsic characteristics of automation and industrial processes, as well as the incorporation of the IIoT concept into the industry. All these features should be considered, as they are fundamental to the adequate operation of diverse processes in the industry. This is because communications are providing increasing support to industrial automation in order to maximize process productivity and automation with minimal human intervention. Once these characteristics are described, how they can be addressed through the use of SDN will be analyzed in Section 3.

One of the general characteristics required by industrial communication networks is a deterministic behavior, which depends on the correct operation of the industrial automation processes. The need for this type of behavior directly influences the design, configuration, and management of the communication network.

The networks of the current industry should consider that new and varied data formats and protocols need to be supported by a common network infrastructure, and that there is also a need for an easily scalable network that ensures the Quality of Service (QoS) variability and adaptive transmission required by the connected devices. These communication network demands proposed by Chen et al. [26] were caused by the increasing introduction of heterogeneous smart devices as well as the recurrent topology variation in different scenarios. The authors also address some problems associated with the management of networks in the manufacturing industry: (a) the workflow linked to the security of the network is complex, as is fault localization; (b) traditional networks do not effectively support real time manufacturing using the cloud, and; (c) adjusting band width of the network [27] in real time according to the flow demanded by data acquisition is difficult, which leads to a reduced use of the network’s resources. Bizanis and Kuipers [28] also point to the massive connection of heterogeneous equipment mentioned above, emphasizing that this causes an overload in IIoT networks, which reinforces the need for scalable networks.

Wan et al. [29] analyze several features essential in IIoT networks, proposing that it is necessary to: (a) achieve higher flexibility and provide better support to the different smart devices connected, regardless of the signals being wired or wireless, real-time, or delayed; (b) update the communication protocols of each device in traditional networks, and develop new methods for the configuration and management of all kinds of network resources due to the new collaboration techniques required by equipment connected in the smart industry; (c) have a high security level to protect the high commercial value of the data generated, with special emphasis on authentication which is carried out at the network and application levels, which represents a problem for the nodes in IIoT; (d) to improve standardization, since the interconnection between the hardware, software, and network components that intervene in IIoT scenarios is not unified enough to support components from different vendors, this being the reason why standard interfaces that allow for the large-scale development of IIoT are necessary.

Many communication technologies and protocols converge in IIoT scenarios. Each of them needs to contribute to ensure the features mentioned above, as well as a dynamic reconfiguration that improves network robustness [30], the adequate exchange of data flows with different delays and among different devices [31], and punctuality and fault tolerance [32], needs that are, in general, markedly different from the field networks. Another characteristic worth mentioning is that different devices of the industry, including wireless equipment, have an IP connection. The use of some wireless technologies, such as LoRa, NBIoT, WIA-PA, WirelessHART, and ISA100.11a, does not contribute to meeting this expectation due to its incompatibility with IP networks. Some proposals to enable the access of these wireless networks to IPv6 connections in order to overcome this limitation are found in the literature [33].

Continuing with the technologies used in the industry, it should be noted that networks based on Time Slotted Channel Hopping (TSCH), such as WirelessHART, ISA100.11a, or 6TiSCH [34], have achieved more than 99.999% reliability from end to end [35], allowing for the isolation of the flow and management of QoS and ensuring more than one decade of shelf life for the battery. However, these technologies were designed to meet the needs of the industry from one decade ago, without considering compatibility with IP networks or the standardized management of the network and orchestration of resources as a need. The Internet Engineering Task Force (IETF) and the work group 6TiSCH have actively worked on this challenge, designing protocols for combining the performance of industrial solutions with networks compatible with IP. One of the goals of this initiative is to define a completely functional architecture in which a combination of IETF protocols enables the convergence sought over the industrial standard IEEE. Despite considerable efforts from 6TiSCH, these protocols have not been adopted extensively by the industry and therefore there are few cases of practical implementations of 6TiSCH.

All characteristics necessary for current industrial networks that have been mentioned up to this point should be considered despite the introduction of IIoT in the industry. To achieve this, it is important that the design and implementation of IIoT considers the aspects and limitations of traditional industrial networks. In this line, the literature contains some architectures that focus on imitating classic industrial networks [36] or that try to cover the requirements for several industrial uses [37]. From these architectures, other needs of IIoT networks can be identified, such as the support of functions such as preventive maintenance, or the integration of 5G into other existing technologies.

## 3. Use of SDN in IIoT

The previous section described the most important aspects and challenges for networks in this new stage of the industry. This section will analyze possible solutions to these obstacles, focusing on SDN and its use in industrial communications, specifically in IIoT scenarios.

### 3.1. Improvements in Communication Network IIoT through SDN

Some works [38,39,40] addressed the possibility of achieving the flexibility of SDN in Local Area Networks (LANs) in Industry 4.0 from different perspectives, as flexibility is one of the issues commonly mentioned in publications. This characteristic contributes especially to the discovery of devices and is relevant in IIoT scenarios with the probability of being escalated, or that require a high degree of automation in the configuration of new devices and services.

Specific cases of requirements for industrial use are analyzed in [41], focusing on solutions that have employed SDN, which allowed the authors to determine the satisfaction degree of each requirement through this network technology. The conclusions of this work result are interesting as they shed some light on the industrial network requirements that may be satisfied by SDN, for example, independence of network technologies, reconfiguration during runtime, safety, and security. Meanwhile, usability and communication over public networks were confirmed to not be exclusively dependent on the use of SDN. The possibility of using heterogeneous communication technologies and managing the network comprehensively with independence from the technologies used is one of the biggest advantages of SDNs. Additionally, the demonstrated effectiveness of SDNs in IIoT networks allows the latter to benefit from both their ease of implementation and the costs associated with this.

The study in [26] highlights the simplification of management networks that can be achieved using SDN with centralized software control, which simplifies the required hardware. Additionally, the problems described in Section 2 concerning network management in the manufacturing industry require smart management of the network, which can be implemented with the programmability and centralization offered by SDN.

In [28], the possibility of employing SDN for the creation of complex access rules for devices, with the different access levels necessary, is explored. Addressing security as well, Wan et al. [29] propose that the global vision of all the network traffic that can be achieved with SDN allows for developing distributed access control. This also enables the improvement of other security measures compared to traditional networks, which are launched at different management/security modes.

The limitations of many IIoT devices are the reason for their high security vulnerability. Such devices tend to be the target of a variety of attacks, such as Denial of Service (DOS), Man-in-Middle (MIM), or flooding attacks. This becomes more relevant for critical applications such as those used by the industry. SDNs have been employed to tackle some security problems of this nature, particularly at the network level [42].

From the above, the analysis of Distributed Denial-of-Service (DDoS) attacks [43] is fundamental for SDN [44,45]. The fact that every new flow received on the data plane entails sending packages to the controller facilitates this type of attack. The work in [46] conducts a detailed review on this and other vulnerabilities presented by SDNs, as well as solutions developed by the scientific community.

The above-mentioned global view of SDNs is key for management as well. Since the controller keeps information about the network topology and status at any moment, this can be managed in a much more efficient way. Thus, ISA100.11a and WirelessHART, two of the main technologies in Industrial Wireless Sensor Networks (IWSN), have adopted SDN as a centralized routing mechanism [47]. To use SDN as a routing mechanism [48], it is important to reduce the overhead of the packets, and this issue has received attention in the literature [49].

Flexibility is indispensable in new industrial networks, as well as in IIoT as a whole. In [29], achieving this by defining not only the network but the whole IIoT through software is proposed. Regarding the network itself, SDN is used in internal and public networks related to the industry. In this way, the massive update of protocols and methods pertaining to the equipment used in such networks is avoided.

Hu [50] also supports the definition of the main IIoT elements by software. Hu specifically highlights wireless field devices, IIoT gateways, network infrastructure, and cloud services of IIoT sensors. The main challenges on which [50] focuses are reliability, security, scalability, and QoS.

The study in [51] proposes that out of the three sub-domains of Cyber-Physical Systems (CPS)—computation, communication, and control—communication represents an obstacle for CPS evolution due to the current state and characteristics of traditional communication networks. Therefore, Ahmed et al. [51] propose an SDN architecture for industrial automation.

Furthermore, [32] puts forward the need for an SDN framework that, enhanced with real time services, solves the requirements of industrial applications, particularly those related to timelines and fault-tolerance as mentioned in Section 2.

Although SDN has not achieved all the progress expected regarding its own standardization, it is a technology that can help solve the problem of interface heterogeneity for the exchange of information between the different IIoT elements [29]. In this sense, using network virtualization may also be key.

The strict control expected over the behavior of wireless networks in IIoT [52], put forward by Thubert et al. [53], can be achieved using SDN, according to the same authors. It is proposed that a central processing element is achieved through this technology, which allows for adequate planning and, consequently, an almost deterministic behavior in wireless networks. This is relevant, since IIoT scenarios converge in both wired and wireless networks.

Among the significant improvements that SDN can contribute are communications in IIoT scenarios; however, the achievement of adaptive transmission is noticeable because it has been a rarely addressed topic. Table 1 shows several IIoT scenarios where this idea has been implemented. The variables improved are included in the practical application of each proposal, as well as the way in which the validity of each case procedure was verified. It must be noted that only in one of the studies reviewed was other technology integrated with the proposed solution.

Traditional networks are characterized by performing data and control functions in the same network, which causes several limitations. For example, the adjustment of network infrastructure control to the massive incorporation of final systems, virtual machines, and virtual networks in industrial automation scenarios is impaired [54]. In this study, authors propose a new Industrial Control Network based on SDN (SDNICN). Additionally, they ensure that this solution can improve the flexibility and performance of industrial control networks to a great extent, while meeting the intelligence and informatization requirements for the industry of the future.

The multicast technique has proved an efficient communication mechanism for large scale IIoT. An example of this is factory clouds, where distributed factories are integrated and the problem of adopting multicast IIoT at large scale in terms of QoS is addressed. In this case, SDN multicast was used to improve package loss under network congestion, among other aspects [58].

One SDN application has served as support to the creation and dynamic management of distributed ICT (Information and Communication Technology), which is useful for the rapid creation of prototypes [59]. Additionally, the authors indicate that the proposal also contributes to satisfying the needs of Industry 4.0 in the field of scalable, control-reliable platforms.

Among the proposals for using SDN in IIoT, there are several architectures. For example, the architecture in [60], denominated Software Defined Industrial Automation Network (SDIAN), focuses on improving the scalability and efficiency of the network in industrial automation scenarios. Although the use of SDN contributes to the improvement of specific network parameters, employing Raspberry Pi plates to implement the proposed architecture can limit the scalability of the network in certain scenarios.

One proposal in the literature consists of an open-source software architecture based on the SDN controller OpenDaylight [61]. Its main objective was to create an IIoT scenario and then deal with the difficulties of introducing ICTs in the industry. Despite the results obtained, the architecture depends on the use of the OpenDaylight controller, which is a limitation if another SDN controller needs to be employed.

In [62], an industrial network architecture defined by software and based on an SDN architecture is proposed, integrating edge and cloud computing technologies as well.

One of the implicit advantages of using SDN over traditional networks is the economic aspect, because SDN simplifies the hardware necessary for the network, which translates directly into a reduction in costs. 

### 3.2. Integration of SDN into Other Technologies

A number of the articles reviewed proposed a combination of SDN and other technologies that widened the scope of the problems that could be solved through this paradigm. An example of this is the combination with Network Virtualization (NV) [63]. In [28], the joint use of both technologies potentiates the versatility and scalability necessary in IIoT services.

Virtualization can be used between the data and control planes in such a way that SDN controllers work with abstractions of network resources [63]. In this way, the work of controllers becomes simpler and is performed by slices [64], which makes every SDN application focus on its targets, handling only one slice of the network’s physical resources.

Sakic et al. [65] propose using SDN and Network Function Virtualization (NFV) to satisfy several needs of industrial networks so they enjoy the advantages of these two technologies. The proposal itself is based on an architecture called VirtuWind. As a validation method, two cases of industrial systems are mapped into the proposed architecture. The focus of this paper is mainly wind farms. The work in [66] refers to the wind industry as a hypothetical scenario that demands high performance, security, and reliability standards in connection with several features of VirtuWind.

Another architecture that combines SDN and NFV is found in [67], which seeks to improve the shelf life of node batteries in IWSN. Petroulakis et al. [68] also present an industrial network enabled for SDN and NFV, which aims to potentiate reactive security mechanisms. Concretely, the authors deal with a scenario based on a wind farm.

Furthermore, Li et al. [31] propose using SDN and Edge Computing (EC) [69] to tackle the issue of exchanging data flows with different characteristics and requirements. This would overcome the limitations of conventional methods associated with traditional networks. A similar scenario is presented by Muthanna et al. [70], who combine SDN and Edge Computing through fog nodes to deal with applications sensitive to latency.

Another possible match for SDN is Time-Sensitive Networking (TSN), whose functions have a great impact on timing and scheduling characteristics [41]. This combination requires the development of mechanisms for a strict compliance with some needs of industrial networks.

In [71], the authors combine some of the technologies mentioned with SDN and identify different traffic profiles.

## 4. Considerations for the Implementation of a Comprehensive SDN Solution for IIoT

This section addresses several useful elements to design and implement an SDN for diverse IIoT scenarios. First, a set of aspects that should always be considered in any solution was systematized. Then, an architecture with distributed network control is proposed and, finally, several criteria are defined for the selection of an SDN controller.

### 4.1. Aspects to Consider in the Implementation of a Comprehensive SDN Solution for IIoT

The considerations presented are based on the challenges, technical aspects, and other elements identified in the literature and by the authors; in addition, these considerations are aimed at the implementation of a comprehensive SDN solution for IIoT. Although using SDN to solve most difficulties faced by modern and future industrial networks is an excellent option with infinite possibilities, the matter is not as simple as it seems. There are several obstacles for putting SDN into practice in the different industrial communication scenarios, especially under the IIoT paradigm.

First, the architecture on which SDN applications are built needs to be considered, as this should provide support to heterogeneous communication technologies, whether wired or wireless, to cover the complete range of industrial applications. 

One characteristic critical to many of these applications is network reliability. In this sense, when implementing an SDN-based network, ensuring the reliability of the SDN system is fundamental. To this end, controller redundancy is essential. Whether distributed or centralized, some redundancy should exist in the devices in charge of such a task.

Another important factor is associated with the standard to be used in the communication with the control plane, i.e., between controllers and devices. In this line, as proposed in [26], although OpenFlow is not the only standard allowed by the SDN technology, its rules have been widely accepted and it has become one of the most used standards. Thus, the implementation of SDN based on OpenFlow has already been employed in industrial scenarios such as intranets and data centers. The literature also supports OpenFlow. Furthermore, some authors, like Hu [72], address all the requirements for the implementation of OpenFlow/SDN networks in an effective and practical manner. In the architecture proposed by [51] for industrial automation, OpenFlow is also the standard of preference. The article in [32] presents a set of extensions for OpenFlow that enables real-time reservations, a significant advance for delay-sensitive applications.

In [28], the optimization of SDN is proposed for its use in IIoT in general. In addition, to completely define IIoT by software, the authors of [29] put forward the implementation of a control layer with the following functions: (a) monitorization, (b) management and optimization, and c) acquisition, transmission, and processing according to the scope defined.

Occasionally, SDN implementations are restricted in terms of applicability. For example, to achieve the almost deterministic behavior of wireless networks in IIoT presented in [53], there is a central element (SDN controller) that takes all decisions. Therefore, the SDN controller is in charge of establishing routes and assigning time/frequency slots, among other aspects. The limitation associated with this implementation is that it is only applicable to well-established and periodical flows, such as IWSNs. The challenge is to expand this idea to scenarios with heterogeneous flow, dynamic behavior, and that use the same network infrastructure [28].

In [29], some difficulties in the implementation of SDN in IIoT communication systems are addressed, specifically that: (a) the design of a forwarding plane for IIoT defined by software is challenging, because OpenFlow has improved and the commuter’s flow chart has evolved into a multiple structure table with more fields, which makes the forwarding design of SDN more and more complex, (b) due to the high number of network nodes in IIoT, multiple distributed controllers are necessary, which makes the control plane broad and the coordination and interaction between controllers more difficult, (c) since IIoT is a centralized control system where data is constantly forwarded to controllers [73,74,75,76], delays in forwarding and even package losses can occur, and (d) controller architecture is very complex and system stability is difficult to guarantee.

Wan et al. [29] suggest possible solutions to the difficulties above: (a) SDN should not be considered for all IIoT scenarios, but only if the network scale is considerably large or the security strategy and the planning strategy are very complex, (b) hybrid devices that support both SDN and traditional network architectures need to be developed so that when the package arrives at a port the hybrid device recognizes whether this is an SDN or a traditional network, thereby solving the interoperability issue between both, and (c) if a single controller is responsible for bottlenecks in network performance, a distributed control plane needs to be implemented to allocate tasks among controllers, increasing network reliability by means of controller redundancy.

Following this line of reasoning, it should be noted that the larger the network, the more important the use of SDN. Precisely, [77] deals with an industrial network of bigger dimensions, not restricted, for instance, to the network of a specific factory. In such a scenario, the fundamental focus is to use SDN to provide network services based on application needs. With this aim, the Dynamic Data Distribution Service (DDS) is employed, through which a detailed tracking of application traffic and requirements is achieved.

WirelessHART, WebSocket, and Constrained Application Protocol (CoAP) are among the network technologies that can be used in SDN network scenarios in IIoT to develop a software-defined system which is aimed at satisfying the essential requirements of generic IIoT applications, as proposed in [50]. The results of the work conducted in [51] are interesting since the control and data plane division conducted to materialize the proposed SDN architecture selected the PROFINET standard. Therefore, this is another network technology that can be implemented in industrial SDN systems.

To tackle the difficulties in IIoT systems in general, Qin et al. [78] launched an attractive initiative to simplify some operation aspects in IIoT scenarios, moving to the network level the problems and global requirements that may exist. From a practical point of view, the idea of converting some specific requirements of IoT scenarios into network level requirements implies that a central controller translates the service requirements into the network requirements. Examples of network requirements are minimum data rate, maximum tolerable delay, and package loss for each independent flow [78].

The approach in [53] has an objective associated with wireless networks in IIoT, which is to achieve strict control over their behavior. In this way, the use of SDN aims to respond to the requirements of services that offer support to those networks.

A comprehensive SDN solution like the one proposed by the authors would be formed by a set of SDN applications that separately focus on concrete aspects of the operation of IIoT communications. Therefore, the SDN ecosystem proposal would satisfy several demands, for example, network reliability, adaptive transmission, fault tolerance, and real-time traffic. A solution of this type is useful but difficult to implement, although it would be a considerable improvement to the current industrial networks. In addition, the features of SDN could open the door to new network applications or modifications of applications that form the SDN solution.

Implicit difficulties in the development of this proposal can be reduced when considering a set of principles and foundations that guide its implementation. Additionally, the base of the SDN solution to be developed should be a well-defined industrial network over which the set of SDN applications can be systematically consolidated.

Through the revision of several studies on the suitability of SDN for industrial networks, some researchers point to the need of a holistic approach to deal with the heterogeneity of industrial communication networks [41]. This justifies the development of the SDN solution outlined above.

An aspect closely related to the obstacles to overcome is the classification of network traffic by its characteristics and requirements [79]. For example, Li et al. [31] carried out a classification that divided traffic into ordinary and emergent; yet, this is just an example and traffic can be categorized differently. As part of this solution, a parameter composed of other basic network parameters can be implemented in such a way that the priority of packages is considered, as well as the band width needed and the maximum delay allowed. From this starting point, a numerical value would be sought to differentiate traffic originating from diverse industrial elements. The above also translates into several groups of such elements, which will be treated differently by the communication network.

Additionally, related to the SDN-based IIoT architecture, the design of the technologies to be used needs to be considered. SDN would come first, together with virtualization, edge/cloud computing, and big data, among others.

It must be noted that the intrinsic characteristics of SDN, such as centralization and the real-time response requirements for industrial networks, make the combination of SDN with other technologies an aspect to be considered carefully, albeit a dispensable one. One of the objectives behind this idea is to decentralize controller tasks in some way.

Regarding the structure of the network architecture for IIoT, it should be noted that generally in IIoT there is no marked tendency towards a specific topology. Additionally, when modeling an industrial network, several studies show that clustered networks exhibit some advantages in terms of extensibility, flexibility, and centralized management [80]. Therefore, the network topology to be designed should focus on a hierarchical structure with nodes to which clusters formed by other nodes are connected.

There is an aspect that differentiates IIoT from scenarios of a different nature, which is also an essential part of the SDN ecosystem’s behavior. In traditional IIoT, a routing trajectory between two devices is not changed if not optimal. This is not admissible in IIoT, because one of its objectives is to find a comprehensive SDN solution, which is precisely an adaptive transmission. Such an objective depends on the route changes made through jumps in the network, and/or the transmission power of wireless devices. In this way, the requirements of each type of traffic flow are satisfied.

Depending on the size of the industrial network in which the SDN solution is used, the traffic flow to the network core should be considered. A valid proposal would be designing a system for the most critical case in such a way that the solution works with a high traffic load in terms of both control and data. Such work with traffic should seek load balancing so all network decisions are made in a decentralized way. A factor related to the above is that, when searching for effective fault-tolerant solutions in SDN, it must be considered that in the separation between the control plane and data from the communication network, both require attention to faults separately and according to their particularities [81].

A proposal for SDN solutions is focused not only on defining the network, but the system as a whole by software. In this way, the different elements of the system would have a virtual representation, facilitating the control and management of several resources. An example of this approach was developed in [82], in which an SDN-based Restful framework is proposed for IIoT, specifically for modern manufacturing.

A wide variety of SDN solutions centered on security, one of the biggest challenges for IIoT in general, are present in the literature [83,84,85,86]. However, when the focus is not directly on security, this tends to be neglected. The decoupling of the control and data planes from the network, a fundamental characteristic of SDN, allows anyone to access the hardware where the control software runs, providing full control over the network. Even if a decentralized controller is implemented, there will always be a main controller that maintains the state of the whole network, so this is still a vulnerable point.

Thus, protecting the network by making the vulnerable points secure should not be overlooked if the goal is to develop a comprehensive SDN solution, as mentioned above. Research that focuses on achieving security in an effective way was conducted by Babiceanu and Seker [87]. In addition, Radanliev et al. show how risks can be minimized in IIoT by using automation and Artificial Intelligence (AI) [25].

For larger industrial networks that have traffic sensitive to delay, the QoS offered to devices needs to address this demand. The loading balance in the network plays an essential role in avoiding bottlenecks that cause inadmissible delays.

As indicated in [25], several IIoT models and frameworks have been proposed recently, with many of them combining IIoT with other technologies. Additionally, the authors of this article present a review [25] of the initiatives from different countries, which center on different aspects directly related to Industry 4.0. None of these aspects is specially aimed at industrial communication networks or how to address the shortcomings encountered nowadays from a technical perspective, or whether these will be exacerbated in the future. This confirms that, despite the efforts made to integrate new technologies in IIoT scenarios, there is still a great deal of work to do in the field.

To use SDN in industrial applications, optimization mechanisms that ensure the required QoS can be employed. The main goal of using optimization algorithms is to find the most efficient trajectories in the network according to the achievement objectives. An example of this is the L_1 norm Optimization used in [60], which seeks to find the shortest path to optimize latency. 

Bi et al. [88] address the difficulties faced by industrial networks to support the QoS necessary for some critical applications, such as fault-tolerance, advanced control, remote monitoring, and predictive maintenance. It must be noted that due to economic and political reasons, a realistic solution is to implement SDN in industrial networks in an incremental way, instead of replacing completely the traditional industrial routers with commuters enabled for SDN. Therefore, this study comprises a hybrid where OSPF routers, OSPF and OpenFlow switches are integrated, for instance, to ensure that the QoS requirements for industrial services are met.

### 4.2. Simulation and Implementation on SDN-Based IIoT Networks

Regarding the simulations of SDN-based networks, Mininet [89] is usually employed to assess their behavior. In the literature, this software is the most widespread option for tests prior to the final SDN implementation. For example, the architecture proposed in [60] is verified through this software.

Regarding the implementation of SDN in IIoT, an alternative in the literature is the use of Raspberry Pi (RPi) plates. In an experimental verification of the architecture proposed in [60], the authors use RPi as a software-defined controller rather than traditional proprietary Programmable Logic Controllers (PLC). Additionally, RPi plates are also employed as SDN switches in such a publication, which was often found in the literature review conducted.

The implementation for a comprehensive SDN solution should focus on integrating SDNs into IIoT scenarios, considering devices configurable by software and the software currently used in the industry to this end.

Despite the alternatives mentioned, to achieve implementations that are as realistic as possible, the use of dedicated SDN switches is valuable to prevent these switches from being executed by software with elements at different levels of an SDN–IIoT architecture.

### 4.3. Architecture and SDN Controllers for IIoT

To achieve an adequate use of SDN in IIoT, a relevant aspect is the selection of an architecture that defines the structure of any system of this type. Table 2 presents the advantages, disadvantages, and uses of different architectures.

**Figure 1:**
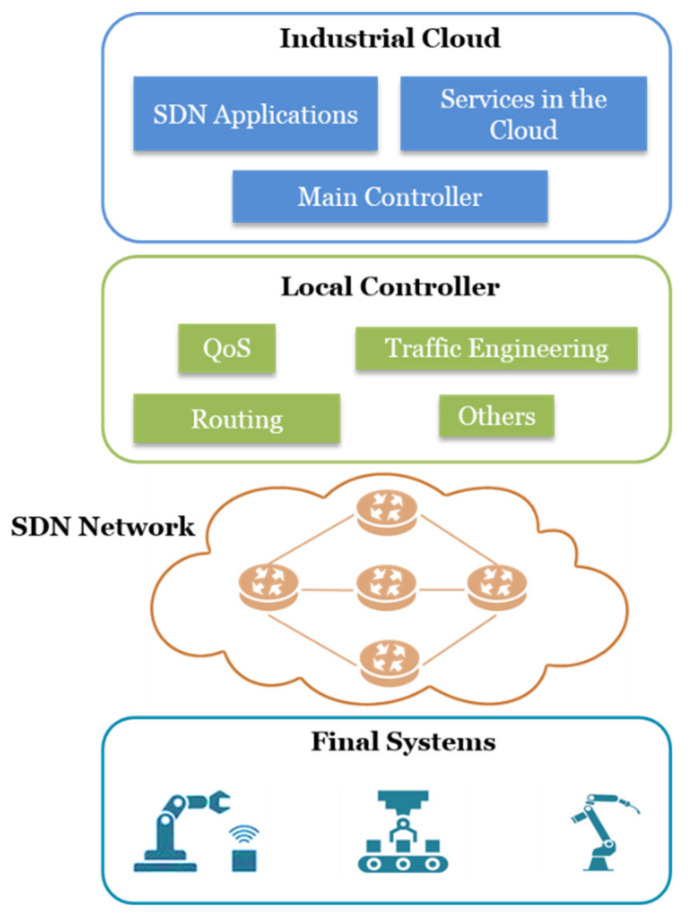
Decentralized architecture for SDN-IIoT solutions.

Considering the specifications in Table 3, the requirements for a network in IIoT, and the advantages of a distributed network control, Figure 1 proposes an architecture with final systems on the lowest layer (first level), which includes all industrial devices and equipment in both traditional machinery and IIoT elements. On a higher layer is the SDN network, composed of the SDN commutators that lack the intelligence of traditional network devices. On the third layer of the architecture is the local controller. In this way, an industrial network that covers a large area in the same locality, or that is geographically distributed, will be able to have different network domains with different requirements. Therefore, every domain will have a different network controller; the load of this controller can be balanced, and this eliminates the single fault-point. Finally, in the industrial cloud layer, a set of services in the cloud is implemented, which are focused mainly on production. Regarding communication, SDN applications that enable the adequate operation of the network are implemented by software at this level. The main controller is included as the most important control element; it corresponds to an SDN controller that has the function of managing the local controllers.

**Figure 2:**
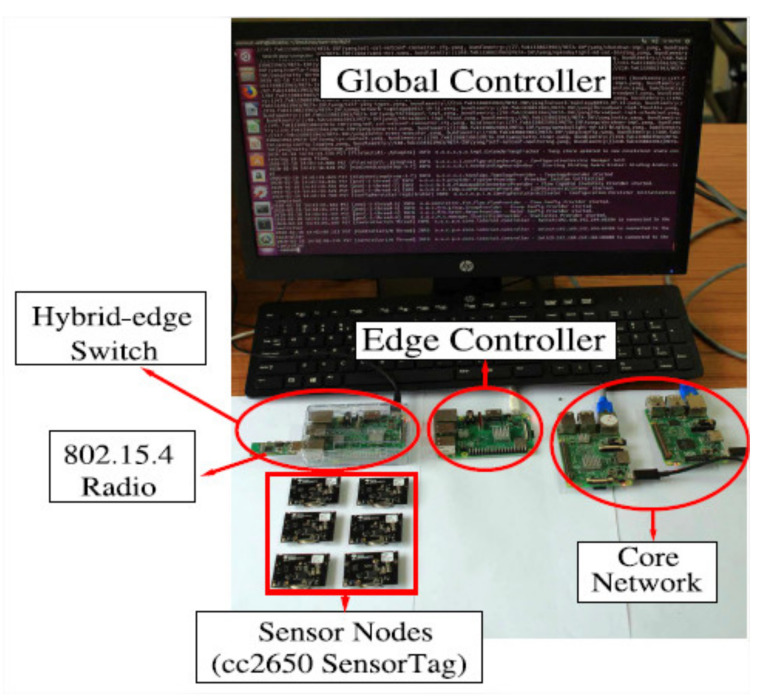
Example of scenario with network control distributed in SDN [93].

Regarding the communication network, the fundamental characteristic of this architecture is the separation of the control plane into secondary elements (local controllers) and a primary element (main controller). In [93], experimental results of a communication network that uses this characteristic of the architecture are shown. As may be seen in Figure 2, the authors design a scenario that employs an edge controller to the local controller and a global controller to the main controller. The use of the edge controller is highlighted as a means for reducing network latency and improving the interoperability range. In this way, considering Figure 2, not all requests from the hybrid-edge switch are sent to the global controller; instead, they receive an immediate response from the edge controller. The global controller only needs to enquire about the action to perform when the edge controller receives an unknown flow for the first time. Network results demonstrate that this scheme contributes mainly to reducing latency and network overhead.

In this way, the industry can remotely attend to two scenarios with different network requirements. An example of this could be a factory with robotized final systems and a smart lighting system located in different geographic areas. Therefore, two local SDN controllers would be employed: one for the factory and another for the location with lighting. In this way, most network requests could be handled locally, reducing traffic circulating towards a third area where the industrial cloud and the main controller would be installed. In the industrial cloud, network, storage, and computing resources would be allocated to execute the SDN Applications programmed by the network administrators. This could include different network policies for the factory and lighting. Finally, cloud services could be dedicated to the control of the robotized final systems of the factory. Currently, some robot control services that have been traditionally conducted locally are being carried out remotely [94]. In this way, the industrial cloud would allocate computing resources to execute software modules with enough logics to control robots remotely. 

One of the critical elements that needs to be carefully selected is the SDN controller; therefore, this topic was dealt with in Section 4.1.

#### SDN Controllers for the IIoT

In SDN, the control plane comprises software-based controllers that control and manage the devices of the underlying data plane and that determine the traffic flows according to the programmed red policies. In this section, the main SDN controllers currently available are presented, which could be useful for IIoT scenarios, ruling out proprietary controllers.

The literature contains diverse works that review and characterize part of the existing SDN controllers. A comprehensive review can be found in [95]; the authors of this article categorize controllers according to the implementation method these allow in the control plane. The main advantages and disadvantages of these methods are summarized in Table 3.

To select a SDN controller, considering different requirements, a full and representative comparative analysis of the existent controllers is necessary. Therefore, it is convenient to assess the controllers of each control plane described in Table 3.

To conduct a selection process, two controllers of each category are considered below, namely, with a centralized control plane (which includes the Ryu and Iris controllers), with a distributed control plane (which includes OpenDaylight and ONOS controllers), and with a hybrid control plane (which includes Fibbing and SDNp controllers). These six controllers were selected based on their good performance. Performance is assessed based on scalability, consistency, reliability, and security, as shown in Table 4. The satisfaction level of each performance parameter is obtained from the results presented in [95].

As seen above, most controllers offer good services for different requirements. Now, from the controllers selected, a specific selection process can be conducted to choose the most suitable SDN controller. To this end, the Analytic Hierarchy Process (AHP) method can be employed, which is designed for assisting in the decision-making process and is widely used in many fields, such as education, manufacturing, politics, engineering, industry, and government [95]. To apply such a method, the following controller characteristics are considered:

- Performance: the performance that a SDN controller may offer is key. In large networks, a controller needs to manage a high number of requests. Therefore, it should be able to do so efficiently to avoid introducing unnecessary delays in the network. The reliability and consistency of the controller could also be considered in its performance assessment.
- Scalability: it is important that networks are scalable in IIoT in general. Therefore, it is desirable that the SDN controller enables the high scalability demanded by a possible massive addition of final systems.
- Load Balancing: when several SDN controllers are used in the network, some of them may be more saturated than others, which could cause delays, package loss, and/or jitter.
- Legacy Network Support: this characteristic is related to one of the three implementation models for the SDN control plane, specifically the hybrid one. In this case, the SDN controller used should be compatible with the traditional network commutators.
- Documentation: this characteristic can be relevant to understanding the operation of a controller and knowing the functions, possibilities, and resources it offers for the development of new SDN applications.
- Modularity: it may be useful for the reutilization of the components of a controller.
- Southbound Interface Support: refers to the versions supported by OpenFlow, as well as to other protocols that broaden the possibilities of the controller.
- Platform: refers to the operational systems in which the controller can be used.
- Virtualization: the options with which the controller can be virtualized, and the possible management of Open vSwitch on the SDN data plane.
- Maturity: the years of maturity of the controller.

The AHP method has been employed for the selection of SDN controllers. An example of the application of this method is presented [96], in which a controller selection process is developed based on an AHP adaptation. Many of the controllers compared were at the initial development stage or had not been extensively tested until the date when this work was conducted. Additionally, a set of selection criteria chosen generically, i.e., not oriented to specific cases, is presented. In [97], another example of the application of the AHP method is found; in this case, the innovation lies in the focus on large campus networks to select the most suitable SDN controller. Specifically, a university network is used under the criterion that this is a big organization.

Analyzing the two works above, it may be concluded that there are three fundamental aspects to consider before applying the AHP mechanism to selecting an SDN controller: (i) which controllers to compare, (ii) what characteristics to assess in order to choose the most suitable controller; and (iii) what priority to assign to each of the characteristics. Depending on the approach taken on these three points, the selection will yield different results oriented to specific cases. Thus, it is important to consider the particularities and requirements of IIoT environments for communication networks before applying the AHP method.

## 5. Conclusions

The review of the current state of research in industrial communications, in which the Industry 4.0 paradigm becomes relevant, leads to the conclusion that traditional network technologies do not satisfy all the requirements of the industry. This article presented a review of a set of works that use SDN to tackle the limitations of traditional networks in the context of IIoT. The approach presented allows for identifying the concrete problems found in the literature. Despite a series of works that offer SDN solutions, none of them is comprehensive enough to cover different industrial uses. This paper is a support tool for developing this type of solution. The review of several limitations as well as their corresponding solutions was complemented with a series of value considerations for integrating comprehensive SDN solutions in IIoT. In addition to these considerations, an architecture with a distributed SDN control network is proposed for such comprehensive solutions. Finally, the basic criteria for the selection of the SDN controller, one of the fundamental elements of the solution, were established.

As for future work, the proposed SDN–IIoT architecture can be complemented with some improvements. The use of NFV is valuable to complement SDN and make it more efficient in some respects. The AHP method will also be employed based on the controllers and characteristics already selected to choose the most suitable controller. Finally, after this selection and with a well-defined architecture, a set of applications will be developed for solving the limitations of current industrial networks.

## Figures and Tables

**Table 1:** Adaptive transmission in IIoT scenarios.

Application Scenarios	Improved Variables	Other Technologies Used	Validation
IIoT [31]	Average delay, throughput, and goodput.	Edge computing	State machine mechanism in MatLab. Metric comparisons with traditional network mechanisms.
Vehicle networks based on 5G [55]	Latency, trunk link throughput, and Bit Error Rate (BER).	-	MatLab simulations.
Vehicle networks defined by software [56]	Average throughput in the network.	-	NS-3 simulations.
Virtualized Wireless networks [57]	QoS, packet loss, and delay.	-	Real scenario with several hosts and FTP and video streaming servers connected to switches and OpenFlow access points.

**Table 2:** Characteristics of different architectures.

Contributions	Advantages	Disadvantages	Use
Plugins IIoT (Plugin IoTDM) [61]	Standard interface for several user applications. Possibility of developing new plugins to connect different technologies for industrial scenarios.	Dependence of main plugin, IoTDM, and therefore of the OpenDaylight controller.	Management and storage of data generated by IIoT devices according to the M2M standard.
Cluster Head [31]	Adoption of network policies according to the traffic behavior in each subsystem.	Possibility of affecting flows due to the general requirements of the sub-system.	Establishment of small subsystems with different requirements through the communication nodes of each cluster.
Edge Computer Server [31]	Reduces the traffic load in the network to the cloud. Contributes to improve response time for time-sensitive services.	Does not substitute cloud servers, the solution is more complex and can be more expensive.	Allocation of computer resources in a sensible way.
Distributed Network Control [90,91,92]	More efficient and rapid control of the different network segments Absence of a single fault-point for an entire industrial network	More complexity in the solution due to the management of several local controllers.	Decentralized management and control of different network segments.

**Table 3:** Advantages and disadvantages of each type of implementation in the control plane.

Implementation of the Control Plane	Advantages	Disadvantages
Centralized	Total visibility and control of the whole network. Simplicity for app developers who should consider the requirements of a single system.	A single controller can deal with bandwidth and control latency issues in large networks. Limitations in terms of scalability and resilience.
Distributed	More robust, scalable, and sensitive to the occurrence of network events such as link faults, new flow requests, intrusion, etc.	Requires a flexible load balance, which implies a control traffic overload in the network. Several interoperability, consistency, controller location challenges, etc.
Hybrid	Allows companies and operators a gradual transition from traditional to SDN networks. Reduces the load on the SDN controller, allowing for a better response and scalability.	Multiple limitations due to the diversity of network devices that convergence in this configuration. Both network configuration, its topology and control are complex.

**Table 4:** Performance of the controllers selected.

Controller	Type of Control Plane	Performance			
		Scalability	Consistency	Reliability	Security
Ryu	Centralized	Medium	Low	High	Low
Iris	Centralized	Medium	Medium	High	Low
OpenDaylight	Distributed	High	High	High	High
ONOS	Distributed	High	High	High	High
Fibbing	Hybrid	High	High	High	Low
SDNp	Hybrid	High	High	High	-
